# Sociodemographic and lifestyle correlates of accelerometer-measured 24-hour physical behavior patterns: a cross-sectional analysis of the ELSA-Brasil cohort

**DOI:** 10.1590/0102-311XEN004226

**Published:** 2026-10-26

**Authors:** Wesley de Souza do Vale, Bruno Pereira de Moura, Rosane Harter Griep, Maria da Conceição Chagas de Almeida, Rogério Tosta de Almeida, Marina Campos Araujo, Maria de Jesus Mendes da Fonseca

**Affiliations:** 1 Escola Nacional de Saúde Pública Sérgio Arouca, Fundação Oswaldo Cruz, Fiocruz, Rio de Janeiro, Brasil.; 2 Instituto Oswaldo Cruz, Fundação Oswaldo Cruz, Fiocruz, Rio de Janeiro, Brasil.; 3 Instituto Gonçalo Moniz, Fundação Oswaldo Cruz, Fiocruz, Salvador, Brasil.; 4 Departamento de Saúde, Universidade Estadual de Feira de Santana, Feira de Santana, Brasil.

**Keywords:** Physical Activity, Sedentary Behavior, Sleep, Accelerometry, Sociodemographic Factors, Atividade Física, Comportamento Sedentário, Sono, Acelerometria, Fatores Sociodemográficos, Actividad Física, Conducta Sedentaria, Sueño, Acelerometría, Factores Sociodemográficos

## Abstract

Physical activity, sedentary behavior, and sleep comprise an integrated 24-hour movement continuum that may influence health outcomes. While these behaviors have been studied separately, limited evidence exists on their combined patterns and related sociodemographic and lifestyle factors in middle-income countries like Brazil. The objective was to evaluate associations between sociodemographic and lifestyle correlates and accelerometer-measured 24-hour physical behavior patterns among Brazilian middle-aged and older adults. This cross-sectional analysis included 9,520 adults (mean age 59.2 ± 8.6 years) from the third wave of the ELSA-Brasil cohort study. Physical activity, sedentary behavior, and sleep were objectively measured using triaxial hip-worn accelerometers for seven consecutive days. Behavioral clusters were defined according to daily time spent in physical activity, sedentary behavior, and sleep using Partitioning Around Medoids cluster analysis. Sociodemographic and lifestyle factors were assessed via standardized questionnaires. Multinomial logistic regression models evaluated associations between correlates and behavioral clusters, with Cluster 3 as the reference category. Three clusters were identified: 1 (lower physical activity, higher sedentary behavior, shorter sleep), 2 (intermediate physical activity, intermediate sedentary behavior, intermediate sleep), and 3 (higher physical activity, lower sedentary behavior, longer sleep). Compared with Cluster 3, participants in Cluster 1 were more likely to be male, to have a higher body mass index, to have higher educational attainment, and to have a high income. Similar but weaker associations were observed for Cluster 2. Distinct 24-hour physical behavior patterns among Brazilian adults were associated with sociodemographic and lifestyle factors. These cross-sectional findings emphasize the need for targeted public health strategies promoting healthier 24-hour behavior profiles.

## Abstract (pt)

Atividade física, comportamento sedentário e sono compõem um contínuo integrado de comportamentos de movimento de 24 horas que pode influenciar desfechos de saúde. Embora esses comportamentos tenham sido estudados separadamente, evidências limitadas sugerem combinações de padrões e associações entre fatores sociodemográficos e de estilo de vida em países de renda média como o Brasil. Este estudo buscou avaliar associações entre correlatos sociodemográficos e de estilo de vida e padrões de comportamentos de movimento de 24 horas medidos por acelerômetro em adultos de meia-idade e idosos brasileiros. Essa análise transversal incluiu 9.520 adultos (idade média 59,2 ± 8,6 anos) da terceira leva do estudo de coorte ELSA-Brasil. Atividade física, comportamento sedentário e sono foram medidos objetivamente por acelerômetros triaxiais afixados ao quadril dos participantes por sete dias consecutivos. Agrupamentos comportamentais foram definidos de acordo com o tempo diário gasto em atividade física, comportamento sedentário e sono usando análise de agrupamentos através de *Partitioning Around Medoids*. Fatores sociodemográficos e de estilo de vida foram avaliados por questionários padronizados. Modelos de regressão logística multinomial avaliaram associações entre correlatos e agrupamentos comportamentais, usando o Agrupamento 3 como categoria de referência. No total, três agrupamentos foram identificados: 1 (atividade física mais baixa, comportamento sedentário mais alto, sono mais curto), 2 (atividade física intermediária, comportamento sedentário intermediário, sono intermediário) e 3 (atividade física mais alta, comportamento sedentário mais baixo, sono mais longo). Os participantes do Agrupamento 1 tinham maior probabilidade de serem homens do que os do Agrupamento 3, possuir índice de massa corporal mais alto, maior nível de escolaridade e alta renda. O Agrupamento 2 apresentou associações semelhantes, mas mais fracas. Padrões distintos de comportamentos de movimento de 24 horas entre adultos brasileiros foram associados a fatores sociodemográficos e de estilo de vida. Esses achados transversais enfatizam a necessidade de estratégias específicas de saúde pública que promovam perfis mais saudáveis de comportamentos de movimento de 24 horas.

## Abstract (es)

La actividad física, el comportamiento sedentario y el sueño conforman un continuo integrado de movimiento de 24 horas que puede influir en los resultados de salud. Si bien estos comportamientos se han estudiado por separado, existe poca evidencia sobre sus patrones combinados y los factores sociodemográficos y de estilo de vida relacionados en países de ingresos medios como Brasil. El objetivo fue evaluar las asociaciones entre los factores sociodemográficos y de estilo de vida y los patrones de actividad física durante 24 horas, medidos con un acelerómetro, en adultos brasileños de mediana edad y mayores. Este análisis transversal incluyó a 9.520 adultos (edad promedio de 59,2 ± 8,6 años) de la tercera ola del estudio de cohorte ELSA-Brasil. La actividad física, el sedentarismo y el sueño se midieron de manera objetiva mediante acelerómetros triaxiales colocados en la cadera durante siete días consecutivos. Se definieron grupos de comportamiento según el tiempo diario dedicado a la actividad física, la comportamiento sedentario y el sueño, utilizando el análisis de agrupamiento *Partitioning Around Medoids*. Los factores sociodemográficos y de estilo de vida se evaluaron mediante cuestionarios estandarizados. Los modelos de regresión logística multinomial evaluaron las asociaciones entre los correlatos y los agrupamientos conductuales, tomando el Agrupamiento 3 como categoría de referencia. Se identificaron tres agrupamientos: el 1 (menor actividad física, mayor comportamiento sedentario, menos horas de sueño), el 2 (actividad física intermedia, comportamiento sedentario intermedio, horas de sueño intermedias) y el 3 (mayor actividad física, menos comportamiento sedentario, más horas de sueño). En comparación con el Agrupamiento 3, los participantes del Agrupamiento 1 tenían más probabilidades de ser hombres, tener un índice de masa corporal más alto, tener un mayor nivel educativo e ingresos altos. Se observaron asociaciones similares, aunque menos marcadas, en el Agrupamiento 2. Los distintos patrones de comportamiento físico a lo largo de las 24 horas entre los adultos brasileños se asociaron con factores sociodemográficos y de estilo de vida. Estos hallazgos transversales resaltan la necesidad de estrategias de salud pública específicas que promuevan perfiles de comportamiento más saludables a lo largo de las 24 horas.

## Introduction

Physical activity, sedentary behavior, and sleep constitute fundamental components of a 24-hour movement continuum, reflecting the integrated and interdependent nature of daily physical behaviors that collectively influence health outcomes across the lifespan ^1^. This framework acknowledges that time allocated to one behavior inherently displaces time spent in others within the fixed 24-hour period, underscoring the importance of examining these behaviors holistically rather than in isolation ^2^. The use of accelerometers has advanced the accuracy of quantifying these behaviors, overcoming limitations of self-report methods such as recall bias and social desirability ^3^. Understanding the patterns and relationships among these behaviors provides a more comprehensive perspective on lifestyle factors that impact chronic disease risk and overall well-being ^1^ ^,^ ^4^.

Epidemiological studies increasingly utilize cluster and pattern analyses to capture the complexity and heterogeneity of daily movement behaviors within populations ^3^ ^,^ ^4^. These data-driven approaches identify naturally occurring patterns that integrate physical activity, sedentary time, and sleep, thereby enabling the development of targeted public health interventions addressing multiple health behaviors simultaneously ^3^ ^,^ ^5^. While such integrative analyses have been conducted predominantly in high-income countries, there remains a paucity of research in low- and middle-income countries, where socioeconomic, cultural, and environmental contexts distinctly shape behavior patterns ^6^.

Brazil provides a relevant context for this investigation due to its ongoing epidemiological transition, marked by increasing noncommunicable diseases and persistent social inequalities ^7^ ^,^ ^8^. Although national surveys have documented sociodemographic disparities in behaviors such as leisure-time physical activity and sedentary behavior, these studies primarily relied on self-reported data, limiting the precision of behavioral assessment ^8^. The *Brazilian Longitudinal Study of Adult Health* (ELSA-Brasil) cohort, a large multicenter study of Brazilian adults, offers an opportunity to objectively measure 24-hour physical behavior patterns using accelerometry and to examine their associations with key sociodemographic and lifestyle factors in a middle-income country setting ^9^ ^,^ ^10^.

Previous studies using ELSA-Brasil accelerometry data have described 24-hour movement behaviors and examined their associations with selected health outcomes, including cognitive function and steatotic liver disease ^11^ ^,^ ^12^ ^,^ ^13^. However, these investigations used analytical approaches focused on descriptive movement metrics, time-use composition, or health-outcome associations ^11^ ^,^ ^12^ ^,^ ^13^. To our knowledge, no previous ELSA-Brasil study has used a cluster-based approach to identify accelerometer-derived 24-hour physical behavior patterns and examine their sociodemographic and lifestyle correlates. Therefore, this study contributes original evidence within the Brazilian context by applying a pattern-based approach to objectively measured physical activity, sedentary behavior, and sleep data in a large multicenter cohort.

Understanding these correlates is essential for designing public health strategies and interventions that address multiple health behaviors simultaneously ^5^. Sociodemographic factors such as age, sex, education, and income, together with lifestyle factors including smoking status and body mass index (BMI), have been individually associated with physical activity and sedentary behavior in previous research ^5^ ^,^ ^14^. However, evidence on their combined influence across integrated 24-hour physical behavior patterns remains limited, particularly in Brazilian adults. Based on evidence of social inequalities in health-related behaviors ^5^ ^,^ ^6^ ^,^ ^8^, we hypothesized that less favorable 24-hour physical behavior patterns, characterized by lower physical activity, higher sedentary behavior, and shorter sleep duration, would be associated with sociodemographic and lifestyle characteristics linked to greater behavioral, occupational, or social vulnerability. Given the exploratory and cross-sectional nature of the cluster-based approach, this hypothesis was examined without assuming causal relationships.

Therefore, this study aimed to evaluate the associations between sociodemographic and lifestyle correlates and accelerometer-measured 24-hour physical behavior patterns among middle-aged and older adults from the ELSA-Brasil cohort.

## Methods

### Study design and participants

This was a cross-sectional study using data from participants in the third wave (2017-2019) of the ELSA-Brasil cohort study. The cohort investigates the incidence and risk factors for chronic noncommunicable diseases in a Brazilian population. At baseline (2008-2010), data were collected from 15,105 active and retired employees of both sexes, aged between 35 and 74 years, from six public institutions located in different Brazilian states: Oswaldo Cruz Foundation (Fiocruz; Rio de Janeiro), University of São Paulo (USP), and the Federal Universities of Bahia (UFBA), Espírito Santo (UFES), Minas Gerais (UFMG), and Rio Grande do Sul (UFRGS) ^9^ ^,^ ^10^.

Accelerometry data were first collected during the third wave (2017-2019). For the present analysis, participants were eligible if they had accelerometer data collected during the third examination wave and available measurements for sociodemographic and lifestyle variables. Among the 10,029 participants with accelerometer data available, 509 (5.1%) were excluded due to missing or invalid accelerometry data (n = 193) or missing measurements for sociodemographic and lifestyle variables (n = 316). The final analytical sample comprised 9,520 participants.

Since ELSA-Brasil is a multicenter study, its research protocol was approved by the Ethics Committees of each participating institution, as well as by the Brazilian National Research Ethics Committee ^9^ ^,^ ^10^. Each data collection wave received specific ethical approval (CAAE: 56021516.0.1001.5240; approval number 1.900.315 dated January 27, 2017), and all participants signed an informed consent form.

### Measurement of sedentary behavior, physical activity, and sleep

Physical activity, sedentary behavior, and sleep over a 24-hour period were assessed using ActiGraph wGT3X-BT accelerometers (https://ametris.com/). The wGT3X-BT is a compact device measuring 3.8cm × 3.7cm × 1.8cm and weighing 27g. It contains a triaxial accelerometer that records acceleration along three distinct axes (anteroposterior, longitudinal, and transverse), providing highly reliable and objective measurements of human physical activity ^15^.

Participants were instructed to maintain their usual daily routines and wear the activity monitor secured by an elastic belt on one side of the hip (aligned with the axillary line of the iliac crest) for seven consecutive days ^16^. The device was removed only during aquatic activities or bathing ^14^. All participants received detailed written instructions on the correct use of the device, as well as contact information for the research team.

Raw data were recorded at a sampling rate of 30Hz and analyzed using ActiLife software, version 6.13.5 (https://ametris.com/). The full ELSA-Brasil accelerometer data processing pipeline, including device configuration, ActiLife settings, wear-time validation, sleep analysis, and behavioral classification procedures, has been described in detail elsewhere ^17^. Wear time validation and classification of wakefulness and sleep periods were conducted using validated algorithms ^18^. A valid day was defined as having at least 600 minutes (10 hours) of wake wear time and an accumulated total sleep time exceeding 160 minutes ^16^. Only data from participants with at least four valid days, including a minimum of two weekdays and one weekend day, were included in the analysis ^19^. Accelerometer data recorded across the three axes were combined into a vector magnitude. Time spent in sedentary behavior (< 200 counts/minute^-1^) and physical activity (≥ 200 counts/minute^-1^) was estimated using established cutoff points ^20^ ^,^ ^21^. These vector magnitude cut points were selected because they were validated in older adult populations using hip-worn ActiGraph devices and were consistent with the age distribution of the ELSA-Brasil third-wave sample ^20^ ^,^ ^21^. In the absence of cohort-specific calibration equations, this approach supported consistent classification of waking physical behaviors, although count-based cut points may introduce some misclassification when applied to heterogeneous age groups ^16^ ^,^ ^17^.

Sleep was derived from the Cole-Kripke algorithm for sleep-wake classification in conjunction with the Tudor-Locke algorithm for sleep period detection ^22^ ^,^ ^23^. Sleep time was calculated as the total duration of the main nocturnal sleep episode within a 24-hour period, excluding daytime naps ^22^ ^,^ ^23^. Because the accelerometer was worn at the hip, sleep estimates should be interpreted as algorithm-derived estimates of the main nocturnal sleep period. Although this strategy supports integrated 24-hour behavior assessment using a single device placement, hip-worn accelerometry may overestimate total sleep time and has lower sensitivity for detecting brief nocturnal awakenings and sleep fragmentation than sleep-focused wrist actigraphy or polysomnography ^17^ ^,^ ^22^ ^,^ ^23^ ^,^ ^24^. Mean daily durations (hours/day) for each behavior were calculated as the average of valid days.

It is important to highlight that, in this study, physical activity is defined as any bodily movement produced by skeletal muscles that results in energy expenditure above resting levels, including but not limited to physical exercise ^25^ ^,^ ^26^. Conversely, sedentary behavior is defined as any waking behavior characterized by an energy expenditure of ≤ 1.5 metabolic equivalents (METs) while in a sitting, reclining, or lying posture ^27^. It should be noted that the sedentary behavior assessed does not include daytime sleep. Sleep is defined as a naturally recurring state of rest, characterized by reduced consciousness, decreased responsiveness to external stimuli, and regulated physiological processes ^28^.

### Patterns of daily physical behaviors

Patterns of daily physical behaviors were derived from objective measurements of time spent in physical activity, sedentary behavior, and sleep. Cluster analysis was employed to group participants based on similarities in the daily durations of these behaviors to identify distinct patterns of daily physical behaviors. Clustering is an unsupervised machine learning technique that organizes individuals into groups (clusters) according to their similarities ^29^. This technique is widely used in pattern recognition to identify naturally occurring groups within datasets.

Patterns of daily physical behaviors were identified using the Partitioning Around Medoids (PAM) clustering method, also known as the K-medoids algorithm ^29^. Before clustering, the behavioral variables were standardized using z-scores to bring physical activity, sedentary behavior, and sleep to a common scale. This approach identifies clusters based on actual data points, referred to as medoids, rather than centroids as used in the K-means algorithm. The primary objective of PAM was to minimize the total distance between all data points and their respective medoids. Compared with K-means, PAM is more robust to noise and outliers because it uses medoids rather than means and minimizes the sum of distances to the nearest medoid, whereas K-means minimizes the sum of squared distances to the nearest centroid. Mahalanobis distance was used to account for the covariance structure among physical activity, sedentary behavior, and sleep. The number of clusters was evaluated using the average silhouette width and the within-cluster sum of squares (WSS) criterion. The silhouette method indicated a 2-cluster solution, whereas the WSS curve suggested an elbow at three clusters. Because the 3-cluster solution provided greater substantive interpretability and allowed identification of a distinct intermediate behavioral pattern, in addition to less favorable and more favorable profiles, the 3-cluster solution was retained for the main analyses. Cluster analysis was conducted using the *factoextra* and *cluster* packages in the R statistical programming language, version 2.4.11 (http://www.r-project.org) ^30^ ^,^ ^31^.

### Sociodemographic and lifestyle variables

Sociodemographic and lifestyle variables were selected a priori based on data availability from the ELSA-Brasil cohort and existing evidence regarding their associations with physical behaviors among adults ^5^ ^,^ ^6^. These variables were assessed using standardized questionnaires during face-to-face interviews ^9^. The variables assessed were age (years), sex (male or female), marital status (married or unmarried [separated, divorced, widowed, single, and other]), self-reported race/skin color (White [White and Yellow] or non-White [Black, Mixed-race, and Indigenous]), educational attainment (no higher education or higher education), employment status (active or retired), occupational level (support, intermediate, or higher), per capita income (categorized into quartiles as low [≤ Q1 “1,640 BRL”], lower-middle [> Q1 “1,640 BRL” and ≤ Q2 “3,045 BRL”], upper-middle [> Q2 “3,045 BRL” and < Q3 “4,685 BRL”], and high [≥ Q3 “4,685 BRL”]), BMI (kg/m^2^), and smoking status (current smoker, former smoker, and never smoker).

Self-reported race/skin color was categorized using predefined groups that reflect sociodemographic similarities specific to the Brazilian population ^13^. Individuals who identified as “Yellow” (Asian descent) were grouped with White participants due to their small representation (n = 238; 2.5%) and similar socioeconomic characteristics ^32^. Likewise, Indigenous individuals (n = 76; 0.8%) were analyzed together with Black and Mixed-race participants, given their shared history of social vulnerability in Brazil ^33^. BMI was calculated as weight in kilograms divided by squared height in meters (kg/m^2^), with weight and height measured by trained professionals using standardized techniques and equipment ^9^.

### Statistical analyses

Descriptive statistics were used to summarize and characterize the sample, with results presented as means with standard deviations (SD) for continuous variables and as counts with percentages [n (%)] for categorical variables.

Generalized linear models, appropriate for cross-sectional study designs, were employed to assess the associations between sociodemographic and lifestyle variables and the patterns of daily physical behaviors.

Initially, bivariate multinomial logistic regression analyses were performed using a logit link function, multinomial distribution, and Wald confidence intervals (CIs) to screen for potential sociodemographic (age, sex, marital status, self-reported race/skin color, educational attainment, employment status, occupational level, and per capita income) and lifestyle (BMI and smoking status) correlates associated with daily physical behavior patterns. A 10% significance level was adopted for these exploratory analyses. This more liberal threshold was used only for variable screening, to reduce the risk of excluding potentially relevant correlates from the multiple models at an early exploratory stage.

Variables that showed statistical significance in the bivariate models were included in the multiple multinomial logistic regression models. These multiple models, also based on the logit link function, multinomial distribution, and Wald CI estimation, were used to evaluate the associations between sociodemographic and lifestyle correlates and the patterns of daily physical behaviors. A 5% significance level was adopted for the final model. The model with the lowest Akaike information criterion (AIC), Bayesian information criterion (BIC), and deviance was selected as the best-fitting model. The analyses focused on estimating the main associations between sociodemographic and lifestyle correlates and patterns of daily physical behaviors. Interaction terms were not assessed.

To assess the robustness of the findings, multicollinearity among exposure variables was evaluated using variance inflation factors (VIF) and tolerance values. VIF values greater than 5 or tolerance values less than 0.2 indicated potential multicollinearity. Additionally, bivariate associations between categorical variables were examined using Cramér’s V coefficient, with values above 0.7 indicating a high degree of association. Furthermore, as part of a sensitivity analysis, sociodemographic characteristics were compared between participants included in the final analytical sample and those excluded due to missing data, using t-tests for continuous variables and chi-square tests for categorical variables.

Odds ratios (ORs) were reported as measures of association, with 95% confidence intervals (95%CI). All statistical analyses were performed using the open-source statistical software Jamovi, version 2.4.11 (https://www.jamovi.org), employing the *GAMLj3* module for multinomial modeling.

## Results

### Participant characteristics

Among the 12,636 participants eligible for the third follow-up of the ELSA-Brasil cohort study, 10,335 agreed to wear accelerometers, and 10,029 had accelerometer data available. After data quality and completeness checks, 509 participants (5.1%) were excluded due to device loss, corrupted or unavailable files, insufficient wear time, or missing key sociodemographic and lifestyle information. Thus, the final analytical sample comprised 9,520 participants (92.1% of those who agreed to wear accelerometers).

The mean age of participants was 59.2 years (SD ± 8.6), and the mean BMI was 27.7kg/m^2^ (SD ± 4.8). Women represented 55.3% of the sample, and 63.9% were married. For self-reported race/skin color, 56.2% identified as White. Most participants had completed higher education (66.2%) and were employed (74.2%). Additional sociodemographic and lifestyle characteristics of the total sample and according to the three patterns of daily physical behaviors are presented in Table 1.

**Table 1:** Sociodemographic and lifestyle characteristics of Brazilian adults from the *Brazilian Longitudinal Study of Adult Health* (ELSA-Brasil) cohort, stratified by patterns of daily physical behaviors (n = 9,520).

Characteristics	Total sample	Patterns of daily physical behaviors			p-value
		Cluster 1	Cluster 2	Cluster 3	
Age (years)	59.2 (± 8.6)	60.2 (± 9.3)	59.0 (± 8.5)	58.4 (± 8.0)	< **0.001**
BMI (kg/m^2^)	27.7 (± 4.8)	28.9 (± 5.3)	27.5 (± 4.5)	26.7 (± 4.4)	< **0.001**
Sex					< **0.001**
Male	4,254 (44.7)	1,504 (15.8)	1,681 (17.7)	1,069 (11.2)	
Female	5,266 (55.3)	1,346 (14.1)	2,157 (22.7)	1,763 (18.5)	
Marital status					0.819
Married	6,081 (63.9)	1,833 (19.3)	2,449 (25.7)	1,799 (18.9)	
Unmarried	3,439 (36.1)	1,017 (10.7)	1,389 (14.6)	1,033 (10.9)	
Self-reported race/skin color					< 0.001
White	5,346 (56.2)	1,893 (19.9)	2,073 (21.8)	1,380 (14.5)	
Non-White	4,174 (43.8)	957 (10.1)	1,765 (18.5)	1,452 (15.3)	
Educational attainment					< 0.001
Higher education	6,303 (66.2)	2,208 (23.2)	2,565 (26.9)	1,530 (16.1)	
No higher education	3,217 (33.8)	642 (6.7)	1,273 (13.4)	1,302 (13.7)	
Employment status					0.005
Active	7,060 (74.2)	2,054 (21.6)	2,861 (30.1)	2,145 (22.5)	
Retired	2,460 (25.8)	796 (8.4)	977 (10.3)	687 (7.2)	
Occupational level					< 0.001
Support	2,488 (26.1)	487 (5.1)	972 (10.2)	1,029 (10.8)	
Medium	3,474 (36.5)	937 (9.8)	1,441 (15.1)	1,096 (11.5)	
Higher	3,558 (37.4)	1,426 (15.0)	1,425 (15.0)	707 (7.4)	
Per capita income					< 0.001
Low	2,599 (27.3)	523 (5.5)	1,037 (10.9)	1,039 (10.9)	
Lower-middle	2,144 (22.5)	557 (5.9)	874 (9.2)	713 (7.5)	
Upper-middle	2,401 (25.2)	771 (8.1)	1,007 (10.6)	623 (6.5)	
High	2,376 (25.0)	999 (10.5)	920 (9.7)	457 (4.8)	
Smoking status					0.027
Smoker	832 (8.7)	230 (2.4)	321 (3.4)	281 (3.0)	
Former	2,872 (30.2)	892 (9.4)	1,121 (11.8)	859 (9.0)	
Never	5,816 (61.1)	1,728 (18.2)	2,396 (25.2)	1,692 (17.8)	

BMI: body mass index.

Note: Cluster 1 (lower physical activity, higher sedentary behavior, shorter sleep), Cluster 2 (intermediate physical activity, intermediate sedentary behavior, intermediate sleep), and Cluster 3 (higher physical activity, lower sedentary behavior, longer sleep) represent distinct patterns of daily physical behaviors derived from the combination of physical activity, sedentary behavior, and sleep measures. Continuous variables are presented as means and standard deviations, and categorical variables as counts and percentages. The p-values correspond to bivariate multinomial logistic regression analyses examining the unadjusted association of each correlate with the patterns of daily physical behaviors. A significance level of 10% was adopted for these exploratory analyses. Bold values indicate statistically significant associations.

### Patterns of daily physical behaviors

Cluster analysis identified three distinct patterns of daily physical behaviors, based on the distribution of time spent in physical activity, sedentary behavior, and sleep over a 24-hour period. Cluster 1, labeled “lower physical activity, higher sedentary behavior, shorter sleep”, included participants who accumulated an average of 7.0 hours/day in physical activity, 10.6 hours/day in sedentary behavior, and 6.4 hours/day in sleep. Cluster 2, termed “intermediate physical activity, intermediate sedentary behavior, intermediate sleep”, was characterized by a mean of 9.0 hours/day in physical activity, 8.2 hours/day in sedentary behavior, and 6.8 hours/day in sleep. Cluster 3, referred to as “higher physical activity, lower sedentary behavior, longer sleep”, represented individuals with 10.9 hours/day in physical activity, 5.4 hours/day in sedentary behavior, and 7.7 hours/day in sleep. A visual summary of these cluster profiles is shown in Figure 1.

**Figure 1:**
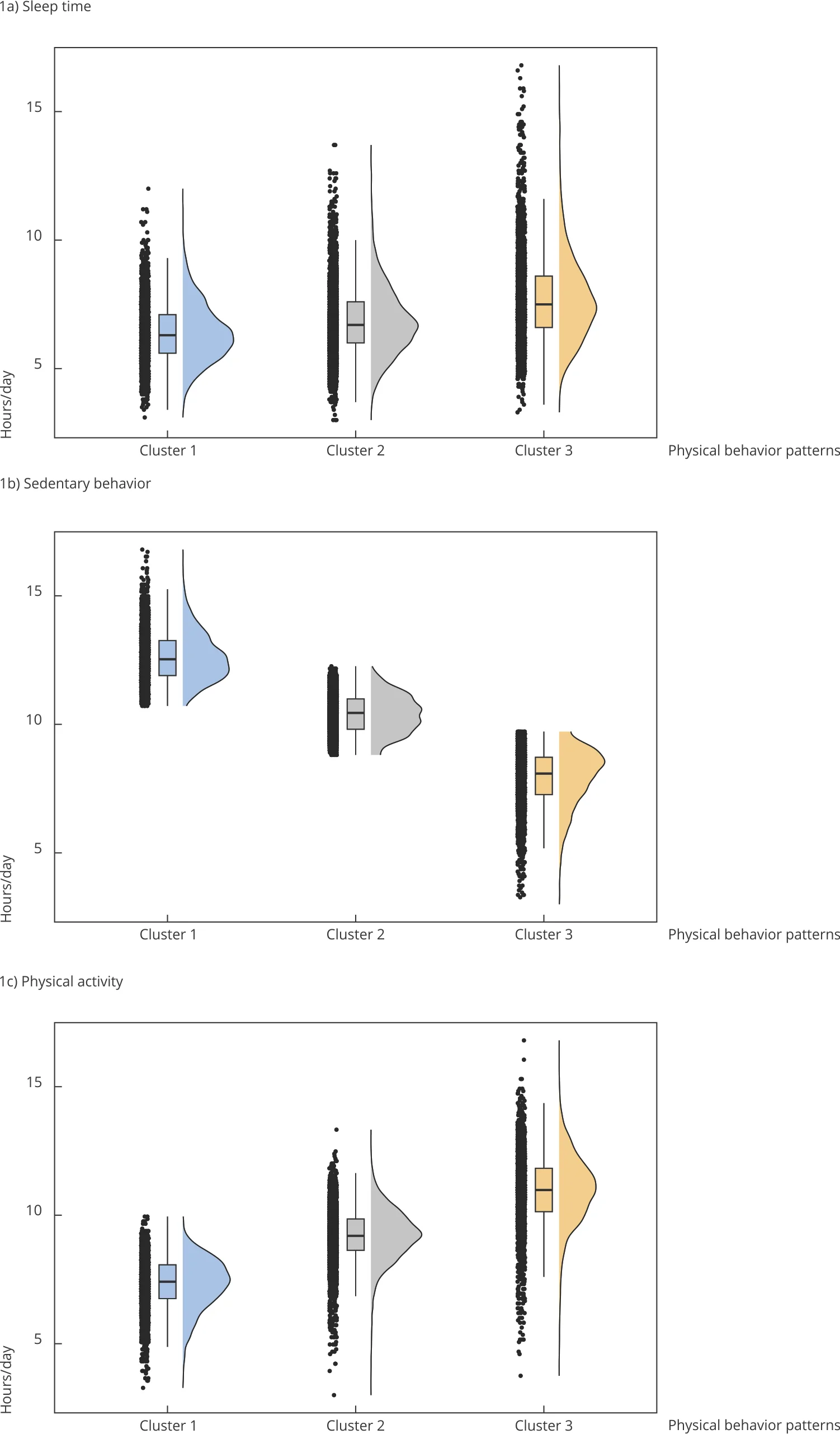
Distribution of sleep time, sedentary behavior, and physical activity across the three identified 24-hour physical behavior patterns in the *Brazilian Longitudinal Study of Adult Health* (ELSA-Brasil) cohort.

Note: violin plots with embedded boxplots and individual observations showing the distribution of daily sleep time, sedentary behavior, and physical activity (hours/day) according to the three clusters identified by Partitioning Around Medoids (PAM) analysis. Cluster 1 represents the pattern characterized by lower physical activity, higher sedentary behavior, and shorter sleep; Cluster 2 represents the intermediate pattern; and Cluster 3 represents the pattern characterized by higher physical activity, lower sedentary behavior, and longer sleep. The figure is intended to facilitate visual comparison of the behavioral profiles identified in the analytical sample.

### Sociodemographic and lifestyle correlates

Initial exploratory analyses using bivariate multinomial logistic regression models indicated significant differences across clusters for age, BMI, sex, self-reported race/skin color, educational attainment, employment status, occupational level, per capita income, and smoking status (Table 1).

Cluster 3 (higher physical activity, lower sedentary behavior, longer sleep) was used as the reference category. Employment status was removed from the multinomial model due to its lack of statistical significance (omnibus test and parameter estimates, p > 0.05), and its exclusion resulted in a reduction in both AIC (-3 points) and BIC (-18 points) (Table 2).

**Table 2:** Multiple multinomial logistic regression analysis of associations between sociodemographic and lifestyle correlates and patterns of daily physical behaviors in Brazilian adults from the *Brazilian Longitudinal Study of Adult Health* (ELSA-Brasil) cohort (n = 9,520).

Response/Effect	Adjusted OR	95%CI		p-value
		Lower	Upper	
Cluster 1 vs. Cluster 3				
Age (years)	1.02	1.01	1.03	< **0.001**
BMI (kg/m^2^)	1.15	1.14	1.17	< **0.001**
Sex				
Male vs. Female	2.30	2.05	2.58	< **0.001**
Self-reported race/skin color				
White vs. Non-White	1.47	1.30	1.66	< 0.001
Educational attainment				
Higher vs. No higher	1.90	1.63	2.23	< 0.001
Occupational level				
Medium vs. Support	1.44	1.23	1.68	< 0.001
Higher vs. Support	1.93	1.57	2.37	< 0.001
Per capita income				
Lower-middle vs. Low	1.23	1.04	1.45	0.017
Upper-middle vs. Low	1.53	1.28	1.83	< 0.001
High vs. Low	2.27	1.86	2.76	< 0.001
Smoking status				
Smoker vs. Former	1.26	1.02	1.57	0.035
Never vs. Former	1.21	1.07	1.38	0.003
Cluster 2 vs. Cluster 3				
Age (years)	1.01	1.01	1.02	0.014
BMI (kg/m^2^)	1.06	1.05	1.08	< 0.001
Sex				
Male vs. Female	1.45	1.31	1.61	< 0.001
Self-reported race/skin color				
White vs. Non-White	1.01	0.91	1.13	0.818
Educational attainment				
Higher vs. No higher	1.38	1.21	1.57	< 0.001
Occupational level				
Medium vs. Support	1.27	1.12	1.45	< 0.001
Higher vs. Support	1.53	1.28	1.83	< 0.001
Per capita income				
Lower-middle vs. Low	1.09	0.95	1.25	0.247
Upper-middle vs. Low	1.28	1.10	1.49	0.001
High vs. Low	1.46	1.22	1.74	< 0.001
Smoking status				
Smoker vs. Former	1.08	0.89	1.30	0.431
Never vs. Former	1.15	1.03	1.29	0.014

95%CI: 95% confidence intgerval; BMI: body mass index; OR: odds ratio.

Noite: Cluster 1 (lower physical activity, higher sedentary behavior, shorter sleep), Cluster 2 (intermediate physical activity, intermediate sedentary behavior, intermediate sleep), and Cluster 3 (higher physical activity, lower sedentary behavior, longer sleep) represent distinct patterns of daily physical behaviors derived from the combination of physical activity, sedentary behavior, and sleep measures. The table presents adjusted ORs, 95%CIs, and p-values from multiple multinomial logistic regression models assessing the associations between sociodemographic and lifestyle correlates and the patterns of daily physical behaviors. Cluster 3 (most favorable pattern) was used as the reference category. A significance level of 5% was adopted. Bold values indicate statistically significant associations.

Results from the multiple multinomial logistic regression models focused on comparisons of Cluster 1 and Cluster 2 relative to Cluster 3. Participants classified in Cluster 1, characterized by lower physical activity, higher sedentary behavior, and shorter sleep, were more likely to be older, to be male, and to have a higher BMI. Higher educational attainment, occupational level, and per capita income were also associated with greater odds of belonging to Cluster 1, with the largest effect observed for the highest income category. In addition, White participants and both current and never smokers had higher odds of belonging to Cluster 1 than their respective reference groups (Table 2).

For Cluster 2, characterized by intermediate physical activity, intermediate sedentary behavior, and intermediate sleep, many of the same correlates remained associated with cluster membership, although the magnitude of association was generally smaller than that observed for Cluster 1. Older age, male sex, higher BMI, higher educational attainment, and higher occupational level were associated with increased odds of belonging to Cluster 2. Associations with per capita income were observed only in the upper-middle- and high-income categories. Unlike the comparison involving Cluster 1, self-reported race/skin color was not associated with Cluster 2 membership. Regarding smoking, only never smokers showed higher odds of belonging to Cluster 2 compared with former smokers (Table 2).

Sensitivity analyses comparing participants included in the final analytical sample (n = 9,520) with those excluded due to missing data (n = 509) showed no statistically significant differences in age, sex, or occupational level (p > 0.05 for all comparisons), suggesting that the excluded participants were broadly comparable to those included. In addition, no evidence of multicollinearity was detected among the exposure variables, and the final model was selected based on the lowest values of the AIC, BIC, and deviance.

## Discussion

Our study identified distinct daily physical behavior patterns among Brazilian adults and demonstrated significant associations between these patterns and key sociodemographic and lifestyle factors, including age, BMI, sex, self-reported race/skin color, educational attainment, occupational level, per capita income, and smoking status. Given the cross-sectional nature of the study, these findings should be interpreted as evidence of association rather than causation. These findings highlight the complex and multifaceted nature of physical behavior patterns and emphasize the need to consider these important correlates when developing targeted public health interventions to promote healthier 24-hour behavior profiles ^1^ ^,^ ^2^.

Our results underscore the presence of sociodemographic gradients in physical behaviors among Brazilian adults. Age showed a modest association, with older adults more likely to belong to Clusters 1 and 2 than to Cluster 3. Previous studies have highlighted age-related declines in physical activity alongside increases in sedentary behavior among adults. The *Canadian Health Measures Survey* found that only 7.1% of Canadian adults meet integrated guidelines, which encompass recommendations for moderate-to-vigorous physical activity, sedentary behavior, and sleep duration, with advancing age associated with a lower likelihood of meeting these guidelines ^34^. Similarly, data from the *Korea National Health and Nutrition Examination Survey* revealed that age was associated with less favorable patterns of 24-hour physical behaviors across different age groups, with the prevalence of the most favorable combinations being only 3.2% in youth, 0.4% in adults, and 0% in older adults ^35^. Sedentary behavior has been identified as an independent predictor of unsuccessful aging in middle-aged and older adults ^36^. However, evidence suggests that replacing sedentary time with light or moderate-to-vigorous physical activity may confer significant benefits for cardiometabolic health and reduce mortality risk ^37^ ^,^ ^38^. These trends highlight the importance of developing age-specific interventions aimed at reducing sedentary lifestyles and promoting active aging.

Elevated BMI was modestly associated with Clusters 1 and 2. This relationship is likely multifactorial. Excess adiposity can limit mobility and reduce exercise capacity, leading to decreased physical activity. Conversely, prolonged sedentary behavior may promote energy imbalance and subsequent weight gain ^39^ ^,^ ^40^ ^,^ ^41^ ^,^ ^42^. Additionally, psychosocial factors such as low motivation, body image concerns, and environmental barriers may also contribute to this cycle, although these were not directly examined in the present study. Some evidence suggests that these associations may be more pronounced in certain subgroups, including older adults and women, underscoring the need for tailored interventions ^43^. The bidirectional nature of this relationship emphasizes the importance of integrated, multidisciplinary interventions that simultaneously address weight management, physical activity promotion, and behavioral change support ^39^ ^,^ ^40^ ^,^ ^41^ ^,^ ^42^. Such approaches are essential to effectively mitigate obesity-related health risks and improve overall physical functioning and quality of life.

Sex differences were also prominent, with males exhibiting a higher likelihood of belonging to Clusters 1 and 2. This finding partially diverges from some studies conducted in Latin America ^6^ and South Korea ^35^, in which females were more frequently associated with less favorable behaviors. However, a study involving Brazilian adults with type 2 diabetes reported that males spent more time in sedentary behavior and less time in light-intensity physical activity compared with females ^14^. Previous evidence suggests that men tend to engage in higher levels of physical activity, particularly in occupational and leisure domains ^44^ ^,^ ^45^, but also spend longer periods in sedentary behaviors throughout the day ^46^. This apparent paradox has been described in other populations and may reflect differences in work-related activities, commuting patterns, and leisure preferences, where men accumulate more total movement but also experience prolonged sedentary episodes ^46^. Occupational and sociocultural contexts within Brazil may further influence these patterns, including potential effects on sleep quality related to evening physical activity and sedentary behavior ^47^ ^,^ ^48^ ^,^ ^49^. These findings reinforce the importance of sex-specific strategies in the design of public health policies and interventions.

Regarding race/skin color, self-identified White participants had a higher likelihood of belonging to Cluster 1. This association may reflect underlying occupational and socioeconomic disparities, as individuals identifying as White in this cohort may be more likely to engage in sedentary occupations or have greater access to lifestyles conducive to sedentary behavior. Such patterns are likely influenced by broader social determinants of health, including disparities in income, education, and work environment, which shape both opportunities and barriers to engaging in physical activity ^50^ ^,^ ^51^ ^,^ ^52^. In the context of this cohort, predominantly composed of public employees, these factors may have contributed to reduced physical activity and increased sedentary time. Addressing such inequalities is essential for developing culturally and contextually appropriate interventions aimed at promoting physical activity and reducing sedentary behavior across diverse groups.

Socioeconomic factors, particularly higher educational attainment, higher occupational level, and greater per capita income, were associated with increased odds of membership in Clusters 1 and 2. Recent studies have explored the complex relationships between socioeconomic status, physical activity, and sedentary behavior. Higher socioeconomic status has been associated with greater engagement in leisure-time physical activity but also with increased sedentary time ^53^ ^,^ ^54^. Paradoxically, higher socioeconomic status groups may exhibit both higher levels of physical activity and greater sedentary behavior, with smaller differences in sitting time across socioeconomic strata ^54^. Previous cluster analyses have revealed distinct patterns of physical activity and sedentary behavior linked to socioeconomic factors, indicating that both socioeconomically disadvantaged and highly advantaged groups may present higher odds of poor sleep health compared with moderately advantaged individuals ^55^. Moreover, occupational physical activity significantly contributes to total physical activity levels, with lower socioeconomic status groups demonstrating higher total physical activity and lower sedentary behavior, whereas higher socioeconomic status groups tend to have lower total physical activity, higher sedentary behavior, and shorter sleep duration ^56^ ^,^ ^57^ ^,^ ^58^.

In the present study, these findings should be interpreted in light of the specific composition of the ELSA-Brasil cohort, which consists of active and retired public servants recruited from six Brazilian capitals ^9^ ^,^ ^10^. This occupational profile likely concentrates individuals with greater job stability, higher educational attainment, and more structured career trajectories than those observed in the general Brazilian population. In addition, many higher-socioeconomic status participants in this cohort may be engaged in administrative, technical, or professional occupations characterized by prolonged sitting, screen-based work, and motorized commuting. Therefore, the association between higher socioeconomic status and less favorable 24-hour physical behavior patterns in this study may reflect the occupational and institutional characteristics of this cohort rather than a generalizable pattern for the Brazilian population as a whole. This interpretation is particularly relevant because the cohort was not designed to represent the full national socioeconomic spectrum ^9^ ^,^ ^10^. These results highlight the importance of understanding the interplay between socioeconomic status, physical activity, sedentary behavior, and sleep, emphasizing that interventions should be tailored to the realities of each socioeconomic group. Such interventions may include workplace strategies to reduce sedentary time among individuals in higher socioeconomic status office-based occupations (e.g., active breaks, sit-stand desks), as well as community-based and environmental approaches to promote active transportation and affordable leisure-time opportunities among lower socioeconomic status populations.

These findings also have potential implications for Brazilian public policies. Within the Brazilian Unified National Health System (SUS, acronym in Portuguese), the promotion of physical activity is already recognized as part of health promotion and primary care practice, and national guidance explicitly recommends that health services and other sectors support more active daily routines ^59^ ^,^ ^60^. In this context, our results suggest that interventions should not focus only on increasing leisure-time physical activity, but also on reducing prolonged sedentary time and improving the overall 24-hour distribution of movement behaviors. For higher socioeconomic status and office-based groups, the workplace appears to be a particularly relevant setting for action, including strategies such as active breaks, support for active commuting, and environmental changes to reduce prolonged sitting. At the same time, within SUS, especially in primary health care, counseling and community-based programs remain essential to ensure that the promotion of healthier movement profiles is aligned with the principles of comprehensiveness and equity ^59^ ^,^ ^60^ ^,^ ^61^.

Smoking status also emerged as an important correlate of daily physical behavior patterns. Former smokers exhibited healthier 24-hour physical behavior patterns compared with current smokers and, to a lesser extent, never smokers. For the comparison between Clusters 1 and 3, both current smokers and never smokers had higher odds of less favorable behavior patterns compared with former smokers, while for Clusters 2 versus 3, this difference was significant only between never and former smokers. One possible explanation is that individuals who quit smoking may adopt broader lifestyle changes, including increased physical activity and reduced sedentary behavior, as part of their overall health improvement efforts ^62^ ^,^ ^63^ ^,^ ^64^. Conversely, the observation that never smokers were also associated with less favorable behavioral patterns could reflect other underlying factors such as comorbidities, psychosocial influences, or unmeasured lifestyle characteristics - including stress levels, sleep disturbances, or dietary habits - warranting further investigation. These findings underscore the potential for smoking cessation programs to integrate physical activity promotion, enhancing overall health outcomes ^62^ ^,^ ^63^ ^,^ ^64^.

An important methodological consideration is that accelerometer-derived estimates may diverge from questionnaire-based findings. Self-reported instruments often emphasize leisure-time physical activity, which tends to be higher among individuals with greater income and educational attainment, while underestimating occupational, transportation, and domestic activities more common in lower socioeconomic groups ^65^ ^,^ ^66^ ^,^ ^67^. In contrast, accelerometry integrates movement across all domains and intensities throughout the entire day, providing a more comprehensive estimate of physical behaviors. Consequently, individuals with lower socioeconomic status may appear less active in questionnaire-based surveys despite higher total activity through work and daily routines, whereas higher socioeconomic status groups may report more leisure-time physical activity but also accumulate greater sedentary time ^66^ ^,^ ^67^ ^,^ ^68^. This methodological distinction helps to explain why accelerometer-derived patterns do not always align with findings from questionnaire-based studies.

This study has several strengths that enhance the validity and relevance of its findings. First, it was conducted with a large, multicenter sample of 9,520 Brazilian adults participating in the third wave of the ELSA-Brasil cohort, one of the largest ongoing epidemiological studies in Latin America. The large sample size increases statistical power and allows investigation of multiple sociodemographic and lifestyle correlates. Second, daily physical behaviors were objectively assessed using triaxial accelerometers, reducing measurement error and recall bias associated with self-reported instruments, although accelerometry also has known limitations (e.g., underestimation of certain activities such as cycling or swimming). Third, the use of cluster analysis enabled identification of distinct, naturally occurring patterns of daily physical behaviors, thereby improving the real-world applicability and interpretability of the findings.

However, some limitations should be acknowledged. The cross-sectional design limits causal inference regarding the directionality of observed associations between sociodemographic and lifestyle correlates and patterns of daily physical behaviors. Nevertheless, cross-sectional studies play a valuable role in generating evidence and identifying associations that can inform future longitudinal research. Additionally, the study population consisted predominantly of civil servants with relatively high education and income levels, which may limit the generalizability of the findings to the broader Brazilian adult population. This sample profile may also have influenced the socioeconomic gradients observed in the present study.

Measurement limitations related to accelerometry should also be considered. Although hip-worn devices are appropriate for assessing physical activity and sedentary behavior in large epidemiological studies, sleep estimates derived from hip placement may overestimate total sleep time and have limited sensitivity for brief nocturnal awakenings or sleep fragmentation. Moreover, although the selected vector magnitude cut points were validated in older adults and were appropriate for the cohort’s age profile, count-based thresholds may introduce some misclassification across heterogeneous age groups. These issues should be considered when interpreting cluster membership. Although a small proportion of participants (5.1%) was excluded from the analytical sample due to missing accelerometry data or exposure variables, sensitivity analyses comparing included and excluded participants revealed no statistically significant differences in key sociodemographic characteristics, such as age, sex, and occupational level. This finding suggests that the risk of selection bias related to missing data was likely minimal. In addition, because the analyses focused on main effects, potential effect modification by sex, age, or socioeconomic position was not assessed.

To our knowledge, this is the first large-scale epidemiological study in Brazil to examine the associations between sociodemographic and lifestyle correlates and patterns of daily physical behaviors, objectively measured by accelerometry, among middle-aged and older adults. These cross-sectional findings expand current evidence and provide a valuable contribution to understanding integrated 24-hour physical behavior patterns in the context of a middle-income country.

## Conclusion

Distinct patterns of daily physical behaviors were identified and were associated with sociodemographic and lifestyle correlates among Brazilian adults. Older age, male sex, higher BMI, White race/skin color, higher educational attainment, higher occupational level, higher per capita income, and smoking status were associated with less favorable patterns (lower physical activity, higher sedentary behavior, and shorter sleep duration). These findings highlight sociodemographic and lifestyle disparities in daily physical behaviors and underscore the importance of developing multifaceted, context-sensitive public health strategies to promote healthier 24-hour physical behavior patterns, particularly among population subgroups at greater risk for unfavorable profiles. Future longitudinal studies are needed to clarify the temporal relationships among these correlates and behavioral patterns and to examine their implications for chronic disease prevention and health promotion. In addition, future studies may explore compositional analytical approaches as a complementary strategy to further examine the interdependent distribution of physical activity, sedentary behavior, and sleep across the 24-hour day.

## Data Availability

The sources of information used in the study are indicated in the body of the article.
